# Personalized Treatment of Patients with Coronary Artery Disease: The Value and Limitations of Predictive Models

**DOI:** 10.3390/jcdd12090344

**Published:** 2025-09-08

**Authors:** Antonio Greco, Davide Capodanno

**Affiliations:** Division of Cardiology, Azienda Ospedaliero-Universitaria Policlinico “G. Rodolico—San Marco”, University of Catania, 95123 Catania, Italy; a.greco90@gmail.com

**Keywords:** acute coronary syndromes, chronic coronary syndromes, coronary artery disease, percutaneous coronary intervention, personalized medicine, predictive models, risk score, risk stratification

## Abstract

Risk prediction models are increasingly used in the management of coronary artery disease (CAD), with applications ranging from diagnostic stratification to prognostic assessment and therapeutic guidance. In the context of CAD and percutaneous coronary intervention, clinical decision-making often relies on risk scores to estimate the likelihood of ischemic and bleeding events and to tailor antithrombotic strategies accordingly. Traditional scores are derived from clinical, anatomical, procedural, and laboratory variables, and their performance is evaluated based on discrimination and calibration metrics. While many established models are simple, interpretable, and externally validated, their predictive ability is often moderate and may be limited by outdated derivation cohorts, overfitting, or lack of generalizability. Recent advances have introduced artificial intelligence and machine learning models that can process large, high-dimensional datasets and identify patterns not apparent through conventional methods, with the aim to incorporate complex data; however, they are not exempt from limitations and struggle with integration into clinical practice. Notably, ethical issues, such as equity in model application, over-stratification, and real-world implementation, are of critical importance. The ideal predictive model should be accurate, generalizable, and clinically actionable. This review aims at providing an overview of the main predictive models used in the field of CAD and to discuss methodological challenges, with a focus on strengths, limitations and areas of applicability of predictive models.

## 1. Introduction

Coronary artery disease (CAD) remains a highly predominant disease, with a global prevalence increasing over the years, up to 2549 per 100,000 subjects in 2019 [1,2,3,4,5,6]. CAD is also a leading cause of mortality, being associated with ~18 million deaths each year worldwide [1,2,3,4,5,6]. In addition, the economic burden of CAD is substantial, with recurrent hospitalizations, invasive procedures, long-term pharmacotherapy, and loss of productivity contributing to significant healthcare costs [7,8,9,10,11].

Different lifestyle, interventional, and pharmacological strategies can be adopted for the prevention and treatment of CAD, aiming to slow down disease progression and minimize the risk of recurrent cardiovascular events [12,13]. However, no universal measures can be established as the optimal management of CAD depends on clinical presentation and the stage of the disease, the individual patient’s characteristics, and the specific clinical, angiographic and socioeconomic circumstances of each case. Indeed, a spectrum of conditions—from asymptomatic atherosclerosis to chronic coronary syndromes (CCS) and acute coronary syndromes (ACS)—are encompassed by the term “CAD”, requiring different management [14,15,16,17,18].

**Figure 1:**
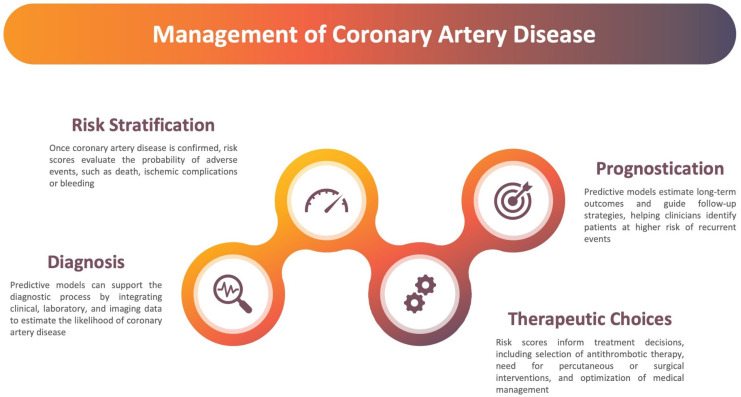
Role of prediction models in the management of coronary artery disease.

In this perspective, a number of strategies have been developed during the last decades in an effort toward personalized medicine, aiming at establishing the optimal management strategy for each individual patient [19,20,21,22,23]. As a consequence, predictive models—ranging from clinical scores to complex machine learning algorithms—have emerged as essential tools to support personalized decision-making (Figure 1) [24,25]. These models integrate data from multiple domains, including demographics, comorbidities, laboratory, imaging, genomics, and proteomics, to stratify patients more accurately and guide therapeutic choices [26]. Yet, they are not exempt from limitations, including issues of derivation, validation, interpretability, easiness of use, freedom from bias, and integration into routine care [27,28,29,30].

The aims of this review article are to provide an overview of the main predictive models used in the field of CAD and to discuss methodological challenges, with a focus on strengths, limitations, and areas of applicability of predictive models. Notably, this review focuses on models that emerged from large trials and/or achieved significant clinical uptake; a comprehensive overview of all available predictive models goes beyond the scope of this review article.

## 2. Risk Stratification and Scores

**Figure 2:**
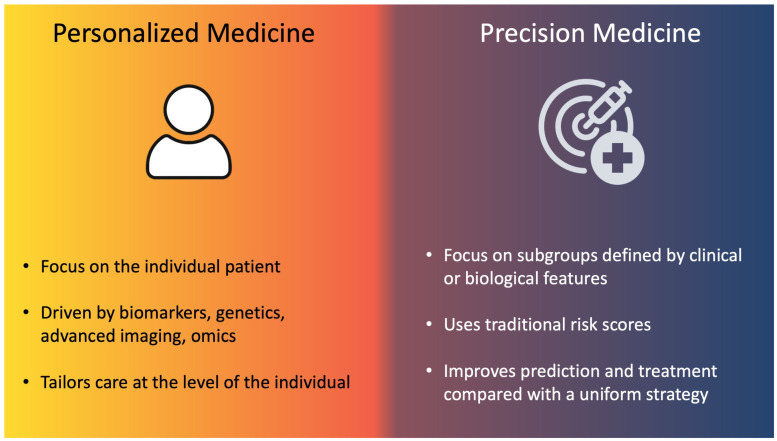
Personalized and precision medicine.

Precision medicine relies on the stratification of patients into subgroups based on shared clinical or biological features, improving prediction and treatment compared with a uniform strategy [31]. Personalized medicine, by contrast, aims to tailor care at the level of the individual, integrating multiple layers of information such as genomic and proteomic profiles, advanced imaging (e.g., plaque characterization, functional imaging), continuous physiologic monitoring through wearables, lifestyle and behavioral factors, and patient preferences (Figure 2) [32]. However, the integration of omics, digital health metrics, and psychosocial context into daily workflows remains limited by cost, complexity, and lack of validated pathways [32].

The concept of quantifying individual cardiovascular risk emerged in the 1970s with long-term cohort studies, including the Framingham Heart Study, which demonstrated that combinations of simple clinical variables (i.e., age, sex, systolic blood pressure, treatment for hypertension, total and high-density lipoprotein cholesterol, diabetes, smoking) could stratify people by their likelihood of developing coronary events [33,34].

By distinguishing high-risk from low-risk patients, the objective of risk stratification is to enable tailored strategies that maximize benefits while minimizing the risks of each treatment by choosing the best treatment for the right patient. Importantly, risk is not a static element, but evolves over time; therefore, an effective risk stratification demands timely repeat assessments [35].

Despite the large number of risk scores developed in the cardiovascular field, their adoption in routine practice has been limited, with several factors potentially contributing to this paradox. Indeed, many models are derived from specific cohorts and show good performance at the population level, but often lack sufficient accuracy and calibration when applied to individual patients in diverse real-world settings. In clinical decision-making, physicians may often prefer direct patient data and clinical judgment over abstract probabilities, particularly when models are not seamlessly integrated into workflows. Furthermore, most scores are static, relying on baseline characteristics and not accounting for the dynamic evolution of disease or the modifying effects of therapy. Indeed, the intensity and type of treatment—ranging from revascularization strategies to the use of evidence-based drugs—can substantially alter an individual’s residual risk during follow-up, with scores derived from baseline characteristics overestimating or underestimating risk if they do not dynamically integrate treatment-related variables.

Key performance metrics for risk models include discrimination and calibration, which jointly represent the extent to which the model correctly categorizes every patient without misclassification [36,37,38,39,40,41]. Discrimination measures the model’s ability to distinguish between patients with and without the event, and it is commonly quantified by the Harrell’s concordance statistics (C-statistics) that correspond to the area under the receiver operating characteristics curve [36]. The C-statistics usually ranges from 0.5 (i.e., no better than chance) to 1.0 (i.e., perfect separation); values above 0.60 are considered sufficient, and >0.75 good [36]. However, it should be noted that such categorization can be acceptable at the population level, while at the individual level even a C-statistics of 0.75 would mean that one case out of four is not correctly classified by the score. Discrimination largely depends on the prevalence and distribution of variables in the population and it is more likely to be low in homogeneous cohorts. However, discrimination is representative of the relative risks among subjects, but is poorly informative about the absolute risk prediction. Conversely, calibration reflects how much the predicted absolute risk estimates are close to the observed estimates across different risk categories [42]. Calibration can be visually assessed or with the Hosmer–Lemeshow goodness-of-fit test [42]. Poor calibration means the model systematically over- or under-estimates risk. Finally, the Brier score is a combined measure of overall accuracy (i.e., mean squared error between predicted probabilities and actual outcomes), with lower Brier scores indicating more accurate probabilistic predictions [36].

**Figure 3:**
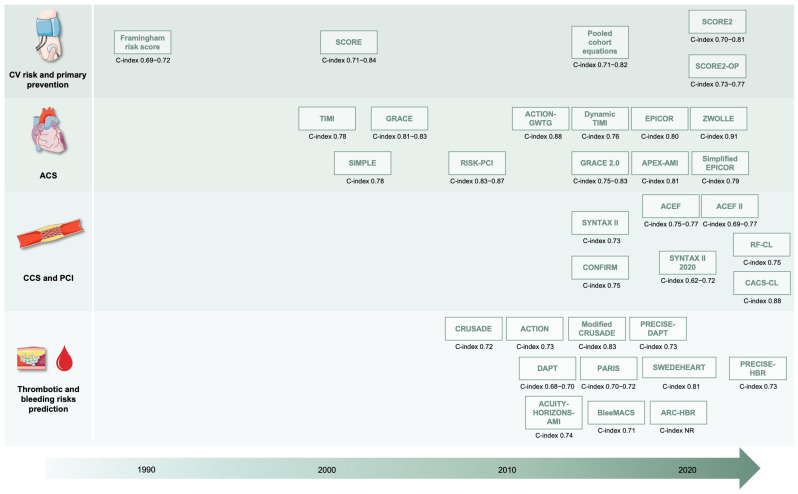
Main predictive models in the field of coronary artery disease. Abbreviations: ACS, acute coronary syndrome; CCS, chronic coronary syndrome; CV, cardiovascular; NR, not reported; PCI, percutaneous coronary intervention.

In the following paragraphs, main clinical risk scores that are relevant to patients with suspected or established CAD will be presented (Figure 3).

### 2.1. Cardiovascular Risk Assessment and Primary Prevention

Risk stratification in primary prevention is essential to identify individuals who may benefit from lifestyle changes or pharmacologic interventions before the onset of overt CAD [43,44,45,46,47]. This is the reason why, although some risk scores estimate the global cardiovascular risk, they are highly relevant for patients with suspected or established CAD (Table 1).

The Framingham risk score was derived from the Framingham Heart Study, a community-based prospective cohort initiated in 1948 [48]. Beyond cardiovascular risk prediction, the risk of coronary artery disease was also investigated. A total of 2489 men and 2856 women aged 30–74 years free of cardiovascular disease at baseline were followed for up to 12 years; during this time, a first coronary event occurred in ~11% of the study participants; using sex-specific Cox proportional hazards models, the investigators identified independent predictors of coronary events (i.e., the composite of death from coronary events, or myocardial infarction [MI]), including age, systolic blood pressure, total cholesterol, high-density lipoprotein cholesterol, smoking, and diabetes, which formed a practical point-based prediction algorithm [49]. In the original cohort, this categorical scoring model showed good predictive accuracy, with C-statistics of 0.69 in men and 0.72 in women [49]. The score was also externally validated in different cohorts, including the large-scale ARIC (Atherosclerosis Risk In Communities) study, confirming moderate-to-good discrimination (C-statistics of 0.69 in men and 0.81 in women) [50]. The Framingham Risk Score was a milestone in cardiovascular risk prediction, as it was one of the first tools to translate longitudinal epidemiological data into individualized estimates. Its easiness of use enabled widespread uptake, but it does not account for social determinants of health, family history, or newer biomarkers. Moreover, its outcome definition (including hard events only) underestimates total cardiovascular burden. Nowadays, the score can be applied using paper charts or online calculators, although current European guidelines recommend SCORE2 (Systematic COronary Risk Evaluation 2), and American guidelines rely on the Pooled Cohort Equations [51,52].

The original SCORE model was developed by the European Society of Cardiology (ESC) to estimate the 10-year risk of fatal cardiovascular events in individuals aged 40–65 years who were free from cardiovascular disease at baseline [53]. It was derived from a pooled dataset of over 250,000 participants from 12 European cohorts, yielding more than 3 million person-years of follow-up and over 7000 fatal cardiovascular events. The model incorporated five key variables (i.e., age, sex, systolic blood pressure, smoking status and total cholesterol or cholesterol/high-density lipoprotein cholesterol ratio) and showed a discrimination between 0.71 and 0.84 across different cohorts [53]. The SCORE model has been implemented in ESC guidelines for cardiovascular prevention via color-coded risk charts and web calculators from 2003 to 2021 [54]. However, the model tended to overestimate the risk in more recent cohorts due to declining cardiovascular mortality [55].The large derivation cohort and geographic calibration made the SCORE widely applicable across Europe. However, by focusing only on fatal events, it may underestimate the total burden of cardiovascular disease, particularly nonfatal MI and stroke. Moreover, it did not consider important risk factors, such as diabetes, family history, or socio-economic status, and its limited age range and calibration based on mortality data from the 1980s and 1990s reduced its relevance in modern populations. These limitations prompted the development of SCORE2 and SCORE2-OP (SCORE2-Older Persons).

SCORE2 was developed using individual-participant data from 45 prospective cohort studies encompassing over 677,000 individuals aged 40–69 years, with 30 million person-years of follow-up and ~30,000 cardiovascular events [56]. SCORE2 was designed to estimate the 10-year risk of both fatal and nonfatal cardiovascular events (i.e., cardiovascular death, MI, or stroke) using competing risk-adjusted Cox regression models; in addition, the model was calibrated separately for four European risk regions (i.e., low, moderate, high, and very high) [56]. The model consists of six key variables (i.e., age, sex, systolic blood pressure, smoking status, total cholesterol and high-density lipoprotein cholesterol) and showed good discrimination in the derivation cohorts, with a C-statistics ranging from 0.70 to 0.81 across age groups and regions. SCORE2 underwent extensive external validation in multiple European populations, confirming its good discrimination [57,58]. SCORE2 replaced SCORE in the 2021 ESC Guidelines on Cardiovascular Disease Prevention and is now the primary recommended model for estimating cardiovascular risk in European adults aged 40–69 [52]. Of note, SCORE2 does not incorporate diabetes as a risk-modifying factor, and its predictive capacity in ethnic minorities or non-European populations remains limited.

SCORE2-OP was developed as an extension of SCORE2 to estimate the risk of individuals aged 70–89 years. It was derived similarly to SCORE2, incorporating competing risk adjustments for non-cardiovascular mortality, which is particularly relevant in older adults; the model was validated in external cohorts and showed acceptable discrimination (C-index ~0.73–0.77) [56,57,59].

The Pooled Cohort Equations were introduced by the American College of Cardiology (ACC) and the American Heart Association (AHA) in 2013 [60]. They were derived from four large, community-based cohort studies in the United States including more than 24,000 participants aged 40–79 years, free of cardiovascular disease at baseline. Cox proportional hazard models were used to estimate the 10-year risk of a first cardiovascular event, with sex- and race-specific equations [60]. Key variables included in these equations include age, sex, race, total and high-density lipoprotein cholesterol, systolic blood pressure, use of anti-hypertensive therapy, diabetes, and smoking status [60].

Despite a good internal discrimination (C-statistics of 0.71–0.82 across the cohorts), external validation studies yielded mixed results, with significant overestimation of risk in modern populations [61]. Limitations of the Pooled Cohort Equations include poor calibration in non-American or multi-ethnic populations and the absence of additional risk factors that emerged in more recent times (e.g., calcium score, high sensitivity, C-reactive protein, lipoprotein (a), genetics) [62].

### 2.2. Acute Coronary Syndrome

In the setting of ACS, risk stratification is crucial for short- and long-term prognostication and treatment decision-making [63,64,65,66,67]. Multiple predictive models have been developed to support risk prediction in these patients (Table 2).

The GRACE (Global Registry of Acute Coronary Events) score was derived from over 100,000 ACS patients, and in its first version encompassed eight variables (i.e., age, Killip class, systolic blood pressure, ST-segment deviation, cardiac arrest at presentation, serum creatinine, elevated cardiac biomarkers, and heart rate) to predict in-hospital death [68]. The score demonstrated strong discrimination, with C-statistics of 0.83 and 0.84 in the derivation and internal validation datasets, respectively, and good performance in external validation in the GUSTO-IIb (Global Use of Strategies to Open Occluded Coronary Arteries-IIb) trial (C-statistics 0.79) [68]. A separate GRACE model was also validated for six-month mortality, showing C-statistics of 0.81 and 0.75 in the derivation and external validation cohorts, respectively [69]. A simplified version using nine items (i.e., age, history of heart failure, peripheral artery disease, systolic blood pressure, Killip class, initial serum creatinine, elevated cardiac biomarkers, cardiac arrest at admission, and ST-segment deviation) was then developed to predict death or MI at six months, with C-statistics of 0.81 (in-hospital death) and 0.73 (death or MI) [70]. This model was externally validated in the GUSTO-IIb trial as well (C-statistics 0.82 for death) [70]. The updated GRACE 2.0 score incorporates nonlinear functions to predict the risk of death or MI at 1 and 3 years, with C-statistics of 0.83 (1-year death), 0.75 (1-year death or MI), and 0.78 (3-year death) [71]. External validation in the FAST-MI (French Registry of Acute ST-Elevation or non-ST-elevation Myocardial Infarction) registry confirmed good discrimination (C-statistics 0.82 for death, 0.78 for death or MI) [71]. Finally, a simplified GRACE 2.0 can be calculated by substituting creatinine with renal failure stage and Killip class with diuretic use [71].

The TIMI (Thrombolysis In Myocardial Infarction) risk score was originally developed for patients with ST-segment elevation MI (STEMI), including eight variables (i.e., age, systolic blood pressure, heart rate, Killip class, ST segment deviation or left bundle branch block, diabetes or history of hypertension or angina, weight, and time to treatment); it showed a C-statistics of 0.78 and 0.75 in the derivation and validation cohorts, respectively, with a more modest discrimination (C-statistics 0.65) when applied to non-ST-segment MI (NSTEMI) [72]. The dynamic TIMI risk score was developed to estimate the 1-year mortality at hospital discharge, including the original eight variables plus six in-hospital complications (i.e., atrial fibrillation, ventricular fibrillation, ventricular tachycardia, renal failure, heart failure, and cardiogenic shock); this score showed good performance in derivation (C-statistics 0.76) and external validation in the TRITON-TIMI 38 (TRial to Assess Improvement in Therapeutic Outcomes by Optimizing Platelet Inhibition with Prasugrel—Thrombolysis In Myocardial Infarction 38) trial (C-statistics 0.81) [73].

The SIMPLE risk index was developed using only three variables (i.e., age, heart rate, systolic blood pressure) collected at first medical contact to predict 30-day mortality; it showed C-statistics of 0.78 for 30-day mortality and of 0.81 and 0.79 for prediction of death within 24 h in the derivation and external validation cohorts, respectively [74].

A large number of prognostic models have been developed to estimate the risk of death or ischemic complications in patients with ACS, using different combinations of clinical, laboratory, and angiographic variables and with varying complexity; these models generally demonstrated good discriminatory performances, but their clinical uptake was heterogeneous (Table 2) [75,76,77,78,79].

### 2.3. Chronic Coronary Syndromes and Percutaneous Coronary Intervention

In the setting of CCS and percutaneous coronary intervention (PCI), risk stratification plays a pivotal role to prevent procedural and long-term complications, to inform therapeutic decisions, and to optimize long-term prognosis (Table 3) [80,81,82,83,84].

The SYNTAX (Synergy Between PCI With Taxus and Cardiac Surgery) score was derived from the SYNTAX trial, enrolling patients with de novo three-vessel and/or left main disease undergoing PCI or coronary artery bypass grafting (CABG); it is an angiographic tool that quantifies coronary lesion complexity based on 12 anatomical features, including bifurcations, chronic total occlusions, severe calcification, and tortuosity [85]. Its primary use was to inform decision-making regarding the optimal revascularization strategy, with higher scores associated with increased risk and worse outcomes after PCI. However, its predictive ability for long-term outcomes is limited and it is influenced by substantial high inter-observer variability.

By integrating clinical prognostic predictors, the SYNTAX score II was developed to estimate four-year all-cause mortality [86]. This model combines the anatomical SYNTAX score with seven clinical variables: age, creatinine clearance, left ventricular ejection fraction, unprotected left main disease, peripheral vascular disease, chronic obstructive pulmonary disease, and female sex, showing good discrimination in the derivation (C-statistics 0.73) and external validation (C-statistics 0.72) cohorts [86]. Recently, the SYNTAX score II has been externally validated in a cohort of patients undergoing coronary artery bypass grafting, showing a C-statistics of 0.73, which was comparable to the performances of EuroSCORE II (European System for Cardiac Operative Risk Evaluation II) (C-statistics 0.73), logistic EuroSCORE (C-statistics 0.74) and ACEF (Age, Creatinine, Ejection Fraction; C-statistics 0.72) [87].

Using the extended 10-year follow-up data from the SYNTAXES (SYNTAX Extension Study) study, the SYNTAX score II 2020 was developed to predict long-term mortality and 5-year major adverse cardiovascular events (MACEs) [88]. The model includes the anatomical SYNTAX score and eight clinical variables (i.e., age, diabetes, creatinine clearance, left ventricular ejection fraction, chronic obstructive pulmonary disease, peripheral artery disease, smoking status, and the presence of three-vessel or left main disease), showing good discrimination for 10-year mortality in either PCI and CABG patients (C-statistics 0.72) and moderate performance for 5-year MACE (C-statistics 0.67 for PCI, 0.62 for CABG).

Another prognostic tool, the CONFIRM score, was derived from the CONFIRM registry, integrating clinical data with findings from computed tomography [89]. The presence of mixed or calcified plaques in proximal segments (C-statistics 0.64) and the number of stenoses >50% in proximal vessels (C-statistics 0.56) were the most relevant predictors of all-cause mortality [89].

The ACEF score was originally developed to predict operative mortality in elective cardiac surgery and was later adapted to PCI patients. The score is simple and includes only three variables (i.e., age, creatinine, left ventricular ejection fraction), while hematocrit and emergency status were added to form the ACEF II score [90]. In the GLOBAL LEADERS trial (n = 15,968), ACEF and ACEF II showed good discrimination for 30-day mortality (C-statistics 0.75 and 0.77, respectively), but modest performance at 2 years (C-statistics 0.72 and 0.69, respectively) [90].

Latest ESC guidelines for the management of CCS introduced the Risk-Factor-weighted Clinical Likelihood (RF-CL) to estimate the pre-test probability of obstructive CAD [14]. This model integrates the number of traditional cardiovascular risk factors with clinical features (i.e., age, sex, symptom characteristics) to better reflect the individual’s likelihood of CAD [14]. Compared to traditional models, the RF-CL approach has shown more appropriate downstream testing recommendations, particularly by identifying patients with very low likelihood in whom diagnostic testing may be safely deferred [91]. Finally, the combination of coronary artery calcium score (CACS) with the RF-CL model (CACS-CL) showed the strongest potential to effectively defer cardiac testing compared with other clinical prediction models or CACS alone [14].

### 2.4. Bleeding and Antiplatelet Therapy Modulation

Antiplatelet therapy is a cornerstone of secondary prevention following ACS or PCI, significantly reducing recurrent ischemic events such as MI and stent thrombosis [92,93,94,95]. However, this therapeutic benefit is counterbalanced by an increased bleeding risk, with bleeding complications associated with adverse prognosis [96,97]. To optimize this trade-off, a proper risk stratification approach is crucial to identify the prevailing risk and the subsequent need for higher- or lower-intensity antiplatelet therapy [98,99,100,101]. Several modulation strategies have been tested [102,103,104,105,106]; in particular, the intensity of dual antiplatelet therapy (DAPT) can be heightened (by increasing DAPT duration or using more potent P2Y_12_ inhibitors) or decreased (by switching to a lower-intensity regimen or shortening DAPT duration) [107,108,109,110,111]. Of note, risk stratification should be intended as a dynamic process, as both the ischemic and bleeding risks may change over time and can be used at different stages of diagnosis and treatment of CAD to inform management and therapeutic strategies (Table 4) [112,113,114,115].

The CRUSADE (Can Rapid Risk Stratification of Unstable Angina Patients Suppress ADverse Outcomes with Early Implementation of the ACC/AHA Guidelines) bleeding risk score was among the first tools specifically developed for predicting in-hospital major bleeding in patients undergoing PCI; it was derived from the CRUSADE registry and included eight baseline clinical and laboratory variables (i.e., systolic blood pressure, heart rate, hematocrit, creatinine clearance, female sex, signs of heart failure at presentation, vascular disease, and diabetes mellitus) [116]. The model demonstrated good discriminatory ability in the derivation (C-statistics 0.72) and internal validation (C-statistics 0.71) cohorts, and was externally validated in NSTEMI (C-statistics 0.82) and STEMI patients (C-statistics 0.80) [116]. A modified version including five variables (i.e., original CRUSADE risk group, P2Y_12_ inhibitor therapy, vascular access, and use of glycoprotein IIb/IIIa inhibitors during and after PCI) was proposed, showing improved discrimination in NSTEMI patients (C-statistics 0.83 and 0.81 in derivation and validation datasets, respectively) [117].

Another bleeding risk score was developed from a pooled cohort of 17,421 ACS patients enrolled in the ACUITY (Acute Catheterization and Urgent Intervention Triage Strategy) and HORIZONS-AMI (Harmonizing Outcomes With Revascularization and Stents in Acute Myocardial Infarction) trials to estimate the 30-day risk of major bleeding, based on six baseline variables (i.e., female sex, advanced age, serum creatinine, white blood cell count, anemia, and ACS type) and the anticoagulation strategy used (heparin plus glycoprotein IIb/IIIa inhibitor vs. bivalirudin alone); the model performed well in the derivation cohort (C-statistics 0.74) [118].

The ACTION bleeding risk score was derived from 90,273 STEMI and NSTEMI cases included in the ACTION Registry-GWTG database to be used at hospital admission to predict in-hospital major bleeding; it included 12 variables (i.e., age, female sex, heart rate, hemoglobin, serum creatinine, electrocardiographic changes, heart failure or shock, diabetes, peripheral artery disease, body weight, systolic blood pressure, and home warfarin use) [119]. The model showed good discrimination in both the derivation (C-statistics 0.73) and validation (C-statistics 0.71) cohorts [119].

The PARIS (Patterns of Non-Adherence to Anti-Platelet Regimen in Stented Patients) score, derived from a cohort of patients undergoing PCI and receiving DAPT, includes two separate models for predicting thrombotic/ischemic and major bleeding events over two years. The bleeding model incorporates six variables (i.e., age, body mass index, current smoking, triple antithrombotic therapy at discharge, anemia, and creatinine clearance <60 mL/min) and showed a good discrimination (C-statistics 0.72) [120].

The PRECISE-DAPT score was derived from a pooled analysis of eight randomized trials including 14,963 patients undergoing PCI; it was developed to estimate out-of-hospital TIMI major or minor bleeding at 12 months following PCI and DAPT [121]. The model, including five variables (i.e., age, creatinine clearance, hemoglobin, white blood cell count, and prior spontaneous bleeding) demonstrated good performance in the derivation cohort (C-statistics 0.73) and was externally validated in both the PLATO trial (C-statistics 0.70) and the Bern PCI Registry (C-statistics 0.66). It has also been incorporated into current ESC guidelines to inform DAPT duration [121].

The BleeMACS score was developed from 15,401 ACS patients undergoing PCI included in the BleeMACS registry, and identifies seven independent predictors of serious spontaneous bleeding at one year (i.e., age, hypertension, vascular disease, history of bleeding, malignancy, creatinine, and hemoglobin); the score performed well in the derivation and internal validation cohorts (C-statistics of 0.71 and 0.72, respectively), but showed reduced discrimination in external validation in the SWEDEHEART registry (C-statistics of 0.65 for PCI and 0.63 for non-PCI patients) [122,123].

Using large data from MI patients in the SWEDEHEART (Swedish Web-system for Enhancement and Development of Evidence-based care in Heart disease Evaluated According to Recommended Therapies) registry, the SWEDEHEART risk model including five variables (i.e., hemoglobin, age, sex, creatinine, C-reactive protein) and one interaction term (hemoglobin*sex) was developed; it showed excellent discrimination (C-statistics 0.81) [124].

The DAPT score was developed to estimate the risk of thrombotic/ischemic versus bleeding events in patients who remained event-free after 12 months of DAPT [125]. It includes nine variables (i.e., age, smoking, diabetes, MI at presentation, prior PCI or MI, use of paclitaxel-eluting stent, stent diameter <3 mm, heart failure or left ventricular ejection fraction <30%, and vein graft PCI), with scores ≥2 indicating net benefit from prolonged DAPT [125]. The score showed C-statistics of 0.70 (ischemic events) and 0.68 (bleeding) in the derivation cohort, but external validation yielded insufficient performances (C-statistics ~0.54 for ischemic events and ~0.49 for bleeding in a nationwide registry) [125].

The Academic Research Consortium for High Bleeding Risk (ARC-HBR) proposed a consensus definition aimed to standardize the identification of patients at high bleeing risk (HBR); a total of 20 criteria were classified as major or minor depending on whether they are associated with a 1-year Bleeding Academic Research Consortium (BARC) type 3 or 5 bleeding risk >4% (major criteria) or >1% (minor criteria); patients with at least one major or two minor criteria are considered HBR [126]. Although no numerical score is provided, a clear stepwise increase in bleeding risk has been observed with a higher number of criteria [126]. The ARC-HBR criteria have been validated in several PCI registries, showing good discrimination for bleeding and thrombotic outcomes (C-statistics ~0.64 for both) [127].

The ARC-HBR trade-off model was developed to quantify the simultaneous risk of MI/stent thrombosis and major bleeding (BARC type 3–5) using eight predictors for each outcome; it showed C-statistics of 0.69 (MI/stent thrombosis) and 0.68 (bleeding) in derivation, and 0.74 for both in external validation in the ONYX ONE trial [128]. The use of a smartphone application enables the simultaneous appraisal of both the risks and their trade-offs.

Among medically managed patients with ACS, the performances of the PRECISE-DAPT (PREdicting Bleeding Complications in Patients Undergoing Stent Implantation and Subsequent Dual Antiplatelet Therapy), PARIS, and DAPT (bleeding component) scores were reasonable and similar to their performances in the derivation PCI populations [129]. Interestingly, there is substantial discordance in the categorization of HBR between the ARC-HBR definition and the PRECISE-DAPT score in patients undergoing coronary stenting; in particular, the PRECISE-DAPT score has been shown to underestimate the bleeding risk [130].

More recently, the PRECISE-HBR score was developed using data from 29,188 patients undergoing PCI from four registries and one trial to convert the ARC-HBR criteria into a weighted score to quantify the bleeding risk [131]. The score consists of seven items (i.e., age, estimated glomerular filtration rate, hemoglobin, white blood cell count, previous bleeding, oral anticoagulation, ARC-HBR criteria) and showed a C-statistics for 1-year BARC 3 or 5 bleeding of 0.73 in the derivation cohort, 0.74 at the external validation in MASTER DAPT (Management of high bleeding risk patients post bioresorbable polymer coated STEnt implantation with an abbReviated versus prolonged DAPT regimen), and 0.73 in the STOPDAPT-2 (Short and Optimal Performance of Dual Antiplatelet Therapy after an Everolimus-Eluting Cobalt-Chromium Stent) trial, with superior discrimination compared with the PRECISE-DAPT and other risk scores [131].

### 2.5. Artificial Intelligence for Risk Prediction

Traditional risk models rely on prespecified variables and assume mostly linear associations between predictors and outcomes, being interpretable and relatively easy to implement but eventually failing to capture higher-order interactions and complex nonlinear relationships. Conversely, models based on artificial intelligence can process large, high-dimensional datasets and identify patterns not apparent through conventional methods [132]. For example, algorithms such as random forest or gradient boosting can handle nonlinearities and interactions without explicit specification, while neural networks can integrate diverse data types such as imaging, genomics, and wearable-derived signals [132].

Over the last few years, artificial intelligence and machine learning have been increasingly used to derive prognostic models (Table 5). In the setting of ACS, the PRAISE (PRedicting with Artificial Intelligence riSk aftEr acute coronary syndrome or Prediction of Adverse Events Following an Acute Coronary Syndrome) model was derived from a cohort of 19,826 patients (80% in the training cohort and 20% in the internal validation cohort) from the BleeMACS (Bleeding Complications in a Multicenter registry of patients discharged with diagnosis of Acute Coronary Syndrome) and RENAMI (REgistry of New Antiplatelets in patients with Myocardial Infarction) registries [133]. Using machine learning techniques, the score was developed to predict one-year all-cause death, MI, and major bleeding, and was also externally validated in 3444 ACS patients from a randomized trial and three prospective registries. The model showed a C-statistics of 0.82 in the internal validation cohort and 0.92 in the external validation cohort for one-year all-cause death, with values of 0.74 and 0.81 for one-year MI, and 0.70 and 0.86 for one-year major bleeding [133]. The AIRE (AI-ECG risk estimation) model was developed using artificial intelligence to predict the risks of all-cause mortality, ventricular arrhythmia, atherosclerotic cardiovascular disease or heart failure directly from standard 12-lead electrocardiograms, showing good predictive ability for all the endpoints (C-statistics between 0.70 and 0.79), also in the external validation dataset (C-statistics between 0.62 and 0.77) [134]. A promising use of artificial intelligence is the merging of multimodal information, such as in a model that incorporated stress cardiac magnetic resonance imaging and coronary computed tomography angiography data to predict MACE in patients with newly diagnosed CAD; the model was imputed with 18 clinical, two electrocardiogram, nine coronary computed tomography angiography, and 12 cardiac magnetic resonance parameters, showing a high predictive ability (C-statistics 0.86), as confirmed in two external validation datasets (C-statistics 0.84 and 0.92) [135].

Recent machine learning applications in CAD span imaging and electronic health records (EHR), increasingly converging in hybrid models. In imaging, deep learning has been applied to coronary computed tomography angiography for automated plaque quantification and non-invasive fractional flow reserve estimation, to invasive angiography and intravascular imaging for lesion characterization, and to echocardiography for chamber quantification and ischemia assessment [136]. EHR-based machine learning models (e.g., gradient-boosted trees and temporal deep learning) integrate demographics, comorbidities, laboratory trajectories, and medications to predict short-term and long-term events [137]. Hybrid strategies typically recalibrate or augment established clinical scores with machine learning features, or ensemble clinical and imaging predictors, with gains most evident in discrimination, calibration, and net benefit when externally validated [138].

A Japanese population-based study applied several machine learning algorithms—including random forest and gradient boosting—to longitudinal data, demonstrating both strong discrimination and excellent calibration [139]. In imaging-driven approaches, a machine learning model combining coronary computed tomography angiography and stress cardiac magnetic resonance outperformed established risk scores in predicting MACE [135]. Furthermore, a prospective cohort of older Chinese patients with CAD and impaired glucose tolerance or diabetes employed gradient boosting to predict one-year mortality, with a C-statistics of 0.836 and good calibration [140].

Interpretability of machine learning models by humans remains a key issue: interpretability tools, such as methods that rank the importance of each variable (e.g., Shapley additive explanations), visualize the effect of changing a single predictor (e.g., partial dependence plots), explore hypothetical “what-if” scenarios, or highlight the most relevant areas in an image (e.g., saliency maps), can improve transparency for clinicians [141]. Ensuring generalizability requires that machine learning models are tested on independent multicenter datasets distinct from those used for development, to confirm that performance is maintained across diverse patient populations and clinical environments. Once implemented, models need continuous surveillance for ‘performance drift’, which is the gradual degradation in accuracy or calibration over time due to changes in patient demographics, clinical practice, or data acquisition methods [142]. Finally, implementation requires adherence to emerging reporting standards and regulatory principles, including documentation of data provenance, risk management, and change-control plans.

Currently, artificial intelligence applications are largely confined to a small number of academic centers, often relying on highly curated and structured datasets that do not reflect the complexity of real-world practice. Moreover, the incremental improvements in predictive performance observed so far—while statistically significant—are often modest when translated into clinical relevance, highlighting that broader validation, accessibility, and integration into daily workflows will be essential before artificial intelligence can meaningfully transform cardiovascular risk prediction. Rather than being confined to isolated academic initiatives, future applications should be built from the outset on broad collaborative networks that also include smaller institutions. This evolution implies moving beyond structured data and predefined variables, toward multimodal integration of unstructured information and advanced techniques such as large language models. A promising path is represented by federated artificial intelligence approaches, in which continuous model training and refinement occur across distributed datasets without centralizing sensitive data [143]. Such an effort will require the active supervision and certification of scientific societies, governmental agencies, and regulatory authorities, ensuring both methodological rigor and third-party validation.

## 3. Strengths and Limitations of Predictive Models

Predictive models can be used with either diagnostic or prognostic purposes; in the field of CAD and PCI, the main applications of risk scores are the estimation of disease probability or severity, the prediction of patient prognosis, and the selection of antithrombotic therapy [36,144,145,146,147]. Models are derived from defined patient cohorts (e.g., registry or trial populations) by selecting candidate predictors and outcomes [148,149]. Predictor variables may be chosen based on clinical expertise or data-driven methods (e.g., stepwise regression, machine learning algorithms) and should be assessed at a defined timepoint (often at presentation or hospital discharge) [82]. Each score can be aimed at detecting a specific outcome of interest, which should be clearly defined (e.g., all-cause mortality, MI, bleeding, or composite events) and measured at a prespecified timepoint [150]. Importantly, variables are drawn from multiple domains (e.g., clinical history, physical examination, laboratory, imaging, angiographic and procedural details, medications), and an optimal trade-off between the implementation of multiple parameters and the easiness of use should be pursued. Notably, it should be kept in mind that a score should be used only if its derivation closely matches the patient scenario (e.g., ACS vs. CCS, or PCI vs. medical management). After derivation, risk prediction models can be internally validated to confirm their performance in a subset of the original population; subsequently, the model should also be tested on another population to ensure its applicability outside the derivation cohort (i.e., external validation) [151,152,153,154,155]. An additional key consideration is the external validation of prediction models across diverse ethnicities, geographies, and clinical settings. Indeed, broader validation in heterogeneous cohorts is essential to ensure generalizability and to avoid disparities in risk assessment when these tools are applied in routine practice.

The applicability of predictive models is often limited by several statistical and practical challenges. Overfitting is a common issue, particularly when models are developed using small or highly selected datasets, leading good performances on training data but failing to generalize to new data. For instance, a study on the atherosclerotic cardiovascular disease (ASCVD) risk score from the American College of Cardiology and the American Heart Association demonstrated that, although the model showed good discrimination and calibration in a Colombian cohort, its performance was less robust in intermediate-risk individuals due to greater heterogeneity in their risk profiles [156]. In addition, calibration is often suboptimal when models are applied to external populations, resulting from differences in patient characteristics between the derivation and validation cohorts. Moreover, spectrum bias refers to the variation in a model performance across different patient populations, often due to differences in disease prevalence or severity; this bias can lead to inaccurate risk predictions if the model is not appropriately validated across the spectrum of the target population.

Beyond statistical concerns, real-world implementation of predictive models faces additional challenges. Data quality and completeness can vary significantly across institutions, affecting the reliability of predictions. In addition, models require periodic retraining to reflect evolving patient populations, therapeutic strategies, and diagnostic practices. Ensuring usability across diverse populations is also critical, as differences in demographics, comorbidities, and healthcare systems can influence both the applicability and acceptance of predictive tools. To mitigate these limitations, strategies such as multicenter external validation, recalibration techniques, and the development of adaptive models are essential.

Notably, predictive models raise important ethical and practical concerns. Equity in model application is critical: algorithmic bias can arise when models are developed from datasets that underrepresent certain demographic or clinical subgroups, potentially leading to systematic under- or over-estimation of risk in these populations [141]. For instance, several risk scores like the Framingham score have been shown to underestimate risk in older adults and particularly in women, while some machine learning models trained predominantly on data from White populations may perform poorly in individuals of African or Asian ethnicity [157]. Over-stratification may also pose risks, as excessively granular risk categories can result in overtreatment or unnecessary patient anxiety [141]. Real-world implementation further introduces challenges. The cost of deploying risk-stratification tools, including software integration and staff training, can limit accessibility, particularly in case of resource constraints. In addition, since incorporation into clinical workflows is essential to ensure adoption by healthcare providers, the lack of user-friendly interfaces and actionable outputs may make models underutilized. For example, models that provide risk estimates without clear guidance on subsequent clinical actions may be ignored by clinicians, reducing their potential benefit [158].

In recent years, artificial intelligence and machine learning approaches have been applied to CAD risk prediction to incorporate larger and more complex data [159,160,161,162,163,164,165]. A meta-analysis of 12 studies of patients with ACS found that machine learning models predicting mortality achieved a higher C-statistics (0.88) than traditional risk scores (0.82) [166]. Although machine learning can improve discrimination, it can bring new challenges, including the “black box” problem (i.e., models sufficiently complex that they are not straightforwardly interpretable to humans), the need for very large high-quality datasets to avoid overfitting, and the possibility that artificial intelligence may inadvertently learn biases present in the data [167].

In addition to statistical challenges related to discrimination and calibration of predictive models, it is essential that they are clinically useful. Key considerations include easiness of use, interpretability, and ability to guide patient management [168]. Indeed, clinicians should favor scores with readily obtainable variables and simple calculation (e.g., integer point scores or availability of online calculators), while models that require dozens of inputs or complex algorithms may not be adopted at the bedside. Interpretability is also important: transparent models (e.g., linear risk equations) allow clinicians to understand how each factor contributes to the overall risk, whereas “black-box” algorithms, such as some machine learning models, may be less intuitive [169,170,171,172]. Of note, a score should have the potential to change care. In summary, an ideal risk score should not only be accurate, but also simple, transparent, and actionable in practice.

The next generation of predictive models in cardiovascular care is likely to move beyond traditional clinical and biomarker inputs, evolving toward integration of diverse data streams that underpin personalized medicine. Genomic and proteomic information, advanced imaging phenotypes, digital health data from wearables, and lifestyle or psychosocial factors may all converge within multimodal algorithms [173]. In addition, the emergence of digital twin technologies—dynamic virtual replicas of patients that assimilate real-time physiological and lifestyle data to simulate disease progression and treatment responses—will offer unparalleled personalization of cardiovascular cares [174]. As such, future models could deliver dynamic, patient-specific predictions that adapt over time. Importantly, incorporating patient preferences and shared decision-making frameworks may help translate statistical predictions into truly individualized care pathways [173]. While these approaches remain aspirational and face challenges related to cost, interoperability and validation, they outline a trajectory from population-level risk stratification toward models that support personalization in cardiovascular medicine.

## 4. Conclusions

Predictive models remain a cornerstone of contemporary cardiovascular medicine, particularly in the management of patients with suspected or established CAD. Over the past decades, a large number of risk scores have been developed to help diagnosis, inform therapeutic decision, and estimate the likelihood of adverse outcomes. More recently, artificial intelligence and machine learning have demonstrated enhanced performance by integrating complex multidimensional data; however, they introduce new challenges related to transparency, validation, and implementation in real-world practice. As the field continues to evolve, future models should balance accuracy with easiness of use, incorporating novel data sources (e.g., imaging, genetics or molecular profiles) while remaining actionable.

## Figures and Tables

**Table 1:** Main prediction models for cardiovascular risk assessment and primary prevention.

Score	Timing of Assessment	Clinical Setting	Predicted Event and Timeframe	Input Variables	C-Statistics	External Validation
Framingham Risk Score	Before disease onset	General population	10-year risk of cardiovascular disease	Clinical: age, sex, systolic blood pressure, treatment for hypertension, smoking status, diabetes, family history of premature cardiovascular disease Laboratory: HDL-C, total cholesterol	0.69 (men), 0.72 (women)	0.61–0.86 in different populations
SCORE	Before disease onset	General population aged 40–65 years	10-year risk of fatal cardiovascular events	Clinical: age, sex, systolic blood pressure, smoking status Laboratory: total cholesterol or cholesterol/HDL-C ratio	0.71–0.84 in different cohorts	0.75 in Spanish without medical history
SCORE2	Before disease onset	General population aged 40–69 years	10-year risk of fatal or nonfatal cardiovascular events	Clinical: age, sex, systolic blood pressure, smoking status Laboratory: total cholesterol, HDL-C	0.70–0.81 across age and regional cohorts	0.64–0.81 in different populations
SCORE2-OP	Before disease onset	General population aged 70–89 years	10-year risk of fatal or nonfatal cardiovascular events	Clinical: age, sex, systolic blood pressure, smoking status Laboratory: total cholesterol, HDL-C	0.73–0.77 across age and regional cohorts	0.59–0.67 in different populations
Pooled Cohort Equations	Before disease onset	General population aged 40–79 years	10-year risk of a first cardiovascular event	Clinical: age, sex, race, systolic blood pressure, use of anti-hypertensive therapy, diabetes, smoking status Laboratory: total cholesterol, HDL-C	0.71–0.82 across sex and race cohorts	0.58–0.71 in different populations

Abbreviations: HDL-C, high-density lipoprotein cholesterol.

**Table 2:** Main prediction models for risk assessment in acute coronary syndrome.

Score	Timing of Assessment	Clinical Setting	Predicted Event and Timeframe	Input Variables	C-Statistics	External Validation
GRACE	Before treatment	ACS	In-hospital and six-month mortality	Clinical: age, heart rate, systolic blood pressure, cardiac arrest at admission, Killip class Laboratory: eGFR, abnormal cardiac enzymes Electrocardiographic: ST-segment deviation	0.83 (in-hospital), 0.81 (6 months)	0.80–0.86 in different populations
GRACE 2.0	At admission or at hospital discharge	ACS	One-year mortality and death or MI, and three-year death	Clinical: age, heart rate, PAD, systolic blood pressure, Killip class, cardiac arrest at admission Laboratory: serum creatinine, elevated cardiac biomarkers Electrocardiographic: ST-segment deviation	0.83 (1-year death), 0.75 (1-year death or MI), 0.78 (3-year death)	0.74–0.81 in different populations
TIMI	Before treatment	STEMI	Thirty-day mortality	Clinical: age, systolic blood pressure, heart rate, Killip class, diabetes or history of hypertension or angina, weight Electrocardiographic: ST-segment deviation or LBBB Procedural: time to treatment	0.78	0.64–0.67 in different populations
SIMPLE risk index	Before treatment	STEMI	Thirty-day mortality	Clinical: age, heart rate, systolic blood pressure	0.78	0.77 in STEMI and NSTEMI
ACTION–GWTG	Before treatment	MI	In-hospital mortality	Clinical: age, heart rate, systolic blood pressure, cardiac arrest at presentation, cardiogenic shock, heart failure Laboratory: eGFR, troponin ratio Electrocardiographic: ST-segment elevation	0.88	NA
ZWOLLE	After treatment	STEMI	Thirty-day mortality	Clinical: age, anterior MI, Killip class Procedural: ischemic time, postprocedural TIMI flow, multivessel disease	0.91	0.72–0.98 in different populations
Dynamic TIMI	Hospital discharge	STEMI	One-year mortality	Clinical: age, systolic blood pressure, heart rate, Killip class, diabetes or history of hypertension or angina, weight Electrocardiographic: ST-segment deviation or LBBB Procedural: time to treatment In-hospital complications: AF, ventricular fibrillation, ventricular tachycardia, renal failure, heart failure, cardiogenic shock	0.76	NA
RISK-PCI	After treatment	STEMI	Thirty-day MACE and mortality	Clinical: age, prior MI, LVEF <40% Laboratory: eGFR, WBC, blood glucose Electrocardiographic: anterior MI, LBBB, third-degree atrioventricular block Procedural: reference vessel diameter ≤2.5 mm, preprocedural TIMI flow 0, postprocedural TIMI flow <3	0.83 (MACE) and 0.87 (death)	0.75–0.87 in STEMI
EPICOR	After treatment	ACS	Two-year mortality	Clinical: age, male sex, education level, BMI, LVEF, quality of life, previous cardiac disease, COPD, no revascularization or thrombolysis, Killip class, diagnosis of STEMI, in-hospital cardiac complications Laboratory: serum creatinine, blood glucose, hemoglobin Therapy: diuretics at discharge, aldosterone inhibitor at discharge,	0.80	0.78 in Asian patients
Simplified EPICOR	After treatment	ACS	Two-year mortality	Clinical: age, male sex, LVEF, quality of life, previous cardiac disease, COPD, no revascularization or thrombolysis, diagnosis of STEMI Laboratory: serum creatinine, blood glucose, hemoglobin	0.79	NA
APEX-AMI	After treatment	STEMI	Ninety-day mortality	Clinical: age, systolic blood pressure, Killip class, heart rate Laboratory: serum creatinine Electrocardiographic: sum of ST-segment deviations, anterior MI	0.81	0.71 in patients with MI

Abbreviations: ACS, acute coronary syndrome; AF, atrial fibrillation; BMI, body mass index; COPD, chronic obstructive pulmonary disease; eGFR, estimated glomerular filtration rate; LBBB, left bundle branch block; LVEF, left ventricular ejection fraction; MACE, major adverse cardiovascular event; MI, myocardial infarction; NA, not available; NSTEMI, non-ST-segment elevation myocardial infarction; PAD, peripheral artery disease; STEMI, ST-segment elevation myocardial infarction; TIMI, thrombolysis in myocardial infarction; WBC, white blood cell count.

**Table 3:** Main prediction models for risk assessment in chronic coronary syndromes and percutaneous coronary intervention.

Score	Timing of Assessment	Clinical Setting	Predicted Event and Timeframe	Input Variables	C-Statistics	External Validation
SYNTAX II	Before treatment, after ICA	PCI or CABG	Four-year mortality	Clinical: age, female sex, LVEF, PAD, COPD Laboratory: serum creatinine Anatomical: SYNTAX score, unprotected left main disease	0.73	0.72–0.73 in different populations
SYNTAX II 2020	Before treatment, after ICA	PCI or CABG	Ten-year mortality and five-year MACE	Clinical: age, diabetes, current smoker, LVEF, PAD, COPD Laboratory: creatinine clearance Anatomical: SYNTAX score, 3-vessel disease or unprotected left main disease	0.72 (10-year death), 0.67/0.62 (5-year MACE for PCI or CABG patients)	0.62–0.72 in different populations
CONFIRM	Before ICA	Suspected CAD	All-cause mortality up to thirty months	Clinical: NCEP ATP III risk Computed tomography: proximal mixed or calcified plaque, proximal stenosis >50%	0.75	NA
ACEF	Before ICA	Patients undergoing elective cardiac operation	Thirty-day and two-year all-cause mortality	Clinical: age, LVEF Laboratory: creatinine	0.75 (30 days), 0.77 (2 years)	0.63–0.79 in different populations
ACEF II	Before ICA	Patients undergoing elective cardiac operation	Thirty-day and two-year all-cause mortality	Clinical: age, LVEF, emergency surgery Laboratory: creatinine, anemia	0.77 (30 days), 0.69 (2 years)	0.70–0.83 in different populations
RF-CL	Before ICA	Suspected CAD	Obstructive CAD	Clinical: age, sex, type of symptoms, family history of CAD, smoking, dyslipidemia, hypertension, diabetes, BMI Laboratory: reduced glomerular filtration rate	0.75	0.78–0.79 in different populations
CACS-CL	Before ICA	Suspected CAD	Obstructive CAD	Clinical: age, sex, type of symptoms, family history of CAD, smoking, dyslipidemia, hypertension, diabetes, BMI Laboratory: reduced glomerular filtration rate Computed tomography: coronary artery calcium score	0.88	0.82–0.86 in different populations

Abbreviations: ATP, adult treatment panel; BMI, body mass index; CABG, coronary artery bypass grafting; CAD, coronary artery disease; COPD, chronic obstructive pulmonary disease; ICA, invasive coronary angiography; LVEF, left ventricular ejection fraction; MACE, major adverse cardiovascular events; NCEP, National Cholesterol Education Program; PAD, peripheral artery disease; PCI, percutaneous coronary intervention; SYNTAX, synergy between percutaneous coronary intervention with taxus and cardiac surgery.

**Table 4:** Main prediction models for assessment of thrombotic and bleeding risks and selection of antithrombotic therapy.

Score	Timing of Assessment	Clinical Setting	Predicted Event and Timeframe	Input Variables	C-Statistics	External Validation
CRUSADE	After PCI	High-risk NSTEMI	In-hospital major bleeding	Clinical: systolic blood pressure, heart rate, sex, signs of heart failure, vascular disease, diabetes Laboratory: creatinine clearance, hematocrit	0.72	0.71–0.81 in different populations
Modified CRUSADE	After PCI	High-risk NSTEMI	In-hospital major bleeding	Clinical and laboratory: CRUSADE score Procedural: puncture pathway Therapy: P2Y_12_ inhibitor therapy, use of GPI during PCI, use of GPI after PCI	0.83	NA
ACUITY-HORIZONS-AMI	After PCI	ACS	30-day major bleeding	Clinical: age, female sex, STEMI or NSTEMI Laboratory: creatinine, WBC, anemia Therapy: heparin plus a GPI or bivalirudin alone	0.74	0.70–0.84 in different populations
ACTION	At the time of PCI	ACS	In-hospital major bleeding	Clinical: age, female sex, heart rate, systolic blood pressure, body weight, heart failure or shock presentation, diabetes, PAD Laboratory: serum creatinine, hemoglobin Electrocardiographic: ECG changes Therapy: warfarin use at home	0.73	0.78 in STEMI
PARIS	After treatment	Patients on DAPT after PCI	Coronary thrombotic events and major bleeding at 2 years	PARIS thrombosis Clinical: diabetes, ACS presentation, current smoking, prior PCI, prior CABG Laboratory: eGFR PARIS bleeding Clinical: age, BMI, smoking status Laboratory: anemia, eGFR <60 mL/min Therapy: triple therapy at discharge	0.70 (thrombotic events), 0.72 (bleeding)	0.59–0.64 in different populations
PRECISE-DAPT	At the time of PCI	ACS and CCS	2-year bleeding	Clinical: age, previous bleed Laboratory: hemoglobin, WBC, eGFR	0.73	0.62–0.70 in different populations
BleeMACS	At the time of PCI	ACS	1-year serious spontaneous bleeding	Clinical: age, hypertension, PAD, previous bleeding, malignancy Laboratory: serum creatinine, hemoglobin	0.71	0.63–0.65 in non-PCI and PCI patients
SWEDEHEART	Before PCI	ACS	In-hospital major bleeding	Clinical: age, sex Laboratory: serum creatinine, hemoglobin, C reactive protein	0.81	0.60 in East-Asian patients
DAPT	After 12 months of uneventful DAPT	Patients on DAPT after PCI	Coronary thrombotic events and bleeding at 12–30 months	Clinical: age, smoking, diabetes, MI at presentation, prior PCI or prior MI, heart failure or LVEF <30% Procedural: paclitaxel-eluting stent, stent diameter <3 mm, vein graft stenting	0.70 (thrombotic events), 0.68 (bleeding)	0.49–0.64 in different populations
ARC-HBR	After PCI	Patients undergoing PCI	BARC type 3 or 5 bleeding at 1 year	Clinical: age, use of long-term oral anticoagulation, spontaneous bleeding requiring hospitalization or transfusion, chronic bleeding diathesis, liver cirrhosis with portal hypertension, long-term use of oral NSAIDs or steroids, active malignancy, previous stroke, intracranial hemorrhage or known brain arteriovenous malformation, nondeferrable major surgery on DAPT, recent major surgery or major trauma Laboratory: eGFR, hemoglobin, platelet count	NR	0.64–0.69 in different populations
PRECISE-HBR	After PCI	Patients undergoing PCI	BARC type 3 or 5 bleeding at 1 year	Clinical: age, previous bleeding, oral anticoagulation, ARC-HBR criteria Laboratory: estimated glomerular filtration rate, hemoglobin, WBC	0.73	0.73–0.74 in different populations

Abbreviations: ACS, acute coronary syndrome; ARC-HBR, Academic Research Consortium—High Bleeding Risk; BARC, Bleeding Academic Research Consortium; BMI, body mass index; CCS, chronic coronary syndrome; DAPT, dual antiplatelet therapy; ECG, electrocardiogram; eGFR, estimated glomerular filtration rate; GPI, glycoprotein IIb/IIIa inhibitor; MI, myocardial infarction; NR, not reported; NSAIDs, non-steroidal anti-inflammatory drugs; NA, not available; NSTEMI, non-ST-segment elevation myocardial infarction; PAD, peripheral artery disease; PCI, percutaneous coronary intervention; STEMI, ST-segment elevation myocardial infarction; WBC, white blood cell count.

**Table 5:** Main prediction models based on artificial intelligence.

Score	Timing of Assessment	Clinical Setting	Predicted Event and Timeframe	Input Variables	C-Statistics	External Validation
PRAISE	At discharge	ACS	All-cause death, MI and major bleeding at 1-year	Clinical: age, LVEF, sex, hypertension, hyperlipidemia, PAD, prior MI, prior CABG, prior stroke, prior bleeding, malignancy, STEMI or NSTEMI, diabetes Laboratory: hemoglobin, eGFR Angiographic or procedural: multivessel disease, complete revascularization, vascular access, drug-eluting stent Therapy: beta-blockers, ACE-inhibitors or ARBs, statins, PPI, OAC	0.82 (death), 0.74 (MI), 0.70 (bleeding)	0.61–0.75 in Asian patients
AIRE	Any	Volunteers, primary and secondary care patients	Mortality risk and time-to-death	Electrocardiogram: AI-based prediction model	0.78	NA
Pezel et al.	Before diagnosis	Symptomatic patients without known CAD referred for CCTA	MACE at up to 7 years	Computed tomography: number of proximal stenoses >50%, number of segments with noncalcified plaques, number of vessels with obstructive CAD Magnetic resonance: number of ischemic segments, number of LGE segments, LVEF	0.86	NA

Abbreviations: ACE, angiotensin converting enzyme; ACS, acute coronary syndrome; AI, artificial intelligence; ARB, angiotensin-receptor blocker; CABG, coronary artery bypass grafting; CAD, coronary artery disease; CCTA, coronary computed tomography angiography; eGFR, estimated glomerular filtration rate; LGE, late gadolinium enhancement; LVEF, left ventricular ejection fraction; MACE, major adverse cardiovascular event; MI, myocardial infarction; NA, not available; NSTEMI, non-ST-segment elevation myocardial infarction; OAC, oral anticoagulant; PAD, peripheral artery disease; PPI, proton pump inhibitor; STEMI, ST-segment elevation myocardial infarction.
